# Supplementary material for: Identification of photoperiod-induced specific miRNAs in the adrenal glands of Sunite sheep (Ovis aries)
Source: Front Vet Sci. 2022 Jul 22;9:888207. doi: 10.3389/fvets.2022.888207 (PMC9354845; doi:10.3389/fvets.2022.888207)
Supplement: Supplementary file 1 [file Data_Sheet_1.ZIP › Supplementary file/miRDeep2 file.zip!/miRDeep2 file/Know_Structure/SP42A1/oar-mir-133.pdf]

miRBase precursor	: oar-mir-133
Total read count	: 311
oar-miR-133 read count	: 306
remaining reads	: 5

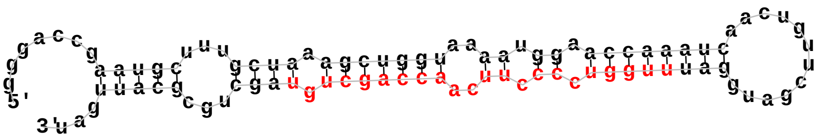

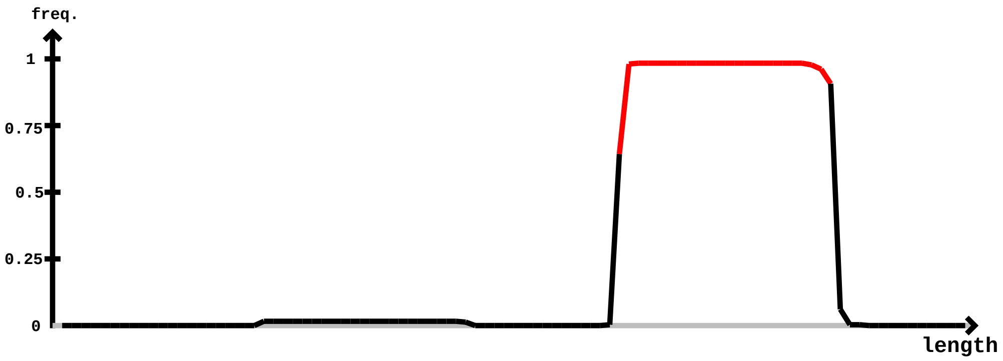

oar-miR-133

```
5'- gggaccgaaugcuuugcuaaagcugguaaaauggaaccaaaucaacuguucgauggauuugguccccuucaaccagcuguagcugcgcauugau   -3'    exp
    .......(((((...((((.(((((((..((.((.((((((((............)))))))).)).))..))))))).))))...)))))...    reads      mm         sample
    ...................agcugguaaaauggaaccaaa............................................................    1          0          seq
    ...................agcugguaaaauggaaccaaau...........................................................    4          0          seq
    .....................................................auuugguccccuucaaccagcugu.............    1          0          seq
    ......................................................uugguccccuucaaccagc................    2          0          seq
    ......................................................uugguccccuucaaccagcu...............    4          0          seq
    ......................................................uugguccccuucaaccagcug..............    13         0          seq
    ......................................................uugguccccuucaaccagcuA..............    2          1          seq
    ......................................................uugguccccuucaaccagcugu.............    172        0          seq
    ......................................................uugguccccuucaaccagcugua............    6          0          seq
    .......................................................ugguccccuucaaccagcu...............    1          0          seq
    .......................................................ugguccccuucaaccagcug..............    2          0          seq
    .......................................................ugguccccuucaaccagcugu.............    89         0          seq
    .......................................................ugguccccuucaaccagcugua............    12         0          seq
    .......................................................ugguccccuucaaccagcuguagc..........    1          0          seq
    ........................................................gguccccuucaaccagcugu.............    1          0          seq
```
